# Was There an Early Habitability Window for Earth's Moon?

**DOI:** 10.1089/ast.2018.1844

**Published:** 2018-08-01

**Authors:** Dirk Schulze-Makuch, Ian A. Crawford

**Affiliations:** ^1^ Center for Astronomy and Astrophysics, Technical University Berlin, Berlin, Germany.; ^2^ School of the Environment, Washington State University, Pullman, Washington, USA.; ^3^ Department of Earth and Planetary Sciences, Birkbeck College, University of London, London, UK.

Our moon is uninhabitable and lifeless today. It has no significant atmosphere, no liquid water on its surface, no magnetosphere to protect its surface from solar wind and cosmic radiation, no polymeric chemistry, and it is subject to large diurnal temperature variations (*e.g.,* Vaniman *et al.,* 1991; Schulze-Makuch and Irwin, 2008). Thus, associating our Moon with habitability seems outrageous, and certainly it would have been just a decade ago. However, results from recent space missions, as well as sensitive analyses of lunar rock and soil samples, have indicated that the Moon is not as dry as previously thought (*e.g.,* Anand, 2010; Hauri *et al.,* 2017). In addition to the probable occurrence of water ice in permanently shadowed polar craters (*e.g.,* Feldman *et al.,* 1998; Baker *et al.,* 2005; Lawrence, 2017), spectroscopic studies also indicate the presence of hydrated surface materials at high, but not permanently shadowed, latitudes (Clark, 2009; Pieters *et al.,* 2009; Li and Milliken, 2017), with evidence for temporal variations over the course of a lunar day (Sunshine *et al.,* 2009). In addition, recent studies of the products of lunar volcanism indicate that the lunar interior also contains more water than was once appreciated and that the lunar mantle may even be as comparably water-rich as Earth's upper mantle (see Hauri *et al.,* 2017, for a review).

The existence of indigenous sources of water implies that the Moon may not always have been as dead and dry as it is today. Insofar as water is required for habitability (*e.g.,* Kasting *et al.,* 1993; although it is not the sole criterion, see Schulze-Makuch *et al.,* 2011), we can speculatively identify two possible windows for lunar habitability. These may have occurred immediately following the accretion of the Moon and some hundreds of millions of years later following outgassing associated with lunar volcanic activity.

Current understanding is that the Moon originated from a gigantic impact 4.5 billion years ago (*e.g.,* Stevenson and Halliday, 2014). The extent to which volatiles were preserved in the Moon-forming debris disk produced by this impact is model-dependent, but impediments to the diffusion of water molecules in a silicate-dominated vapor are expected to result in some water retention in the disk and therefore in the newly formed Moon (Nakajima and Stevenson, 2014; Hauri *et al.,* 2017). The evidence for water concentrations of several hundred parts per million in the mantle source regions of lunar basalts (Hauri *et al.,* 2017; Lin *et al.,* 2017) indicates either that volatiles were indeed preserved during the formation of the Moon or that they were added shortly afterwards by impacting asteroids (*e.g.,* Barnes *et al.,* 2016).

Following accretion, the Moon is expected to have been largely molten, with its silicate components existing in the form of a lunar magma ocean (LMO). Such magma oceans are expected to outgas volatiles, leading to the formation of significant transient atmospheres (Elkins-Tanton, 2008). Indeed, Lin *et al.* (2017) have invoked degassing from the LMO to reconcile the relatively low abundance of water in the post-LMO lunar mantle (at most a few hundred parts per million) with their predictions of much higher values (possibly >1000 ppm) prior to LMO crystallization. On the other hand, some authors have argued that the LMO would have been initially dry following accretion, with the current mantle volatile budget having been added by a subsequent “late veneer” of asteroidal volatiles (*e.g.,* Barnes *et al.,* 2016; Hauri *et al.,* 2017). However, in either case, it appears that significant quantities of water were present in the final stages of LMO evolution. Here, we merely note that outgassing 500 ppm water during the LMO phase (which would be required to bring the higher original values predicted by Lin *et al.* [2017] into agreement with current estimates) could in principle result in a surface water layer of an order of 1 km thickness. Of course, this would be a very optimistic estimate for the depth of any early lunar oceans—water would only be stable at the surface if protected by a sufficiently dense atmosphere, and significant losses would be expected owing to impact erosion (*e.g.,* Melosh and Vickery, 1989)—but it illustrates how much water might potentially have been available.

Needham and Kring (2017) have suggested a second phase of outgassing, and associated peak in lunar atmospheric pressure, as a result of mare basalt eruptions ∼3.5 billion years ago. Gases derived from lava outpourings may have built up an atmosphere of about 10 mbar, which is above the triple-point pressure of water and about 1.5 times the present atmospheric pressure on Mars (and about 3 times as massive as the current martian atmosphere, given the difference in surface gravities). For comparison with the discussion above, Needham and Kring's estimated outgassing of water (∼10^14^ kg) would equate to a global layer having an average depth of ∼3 mm.

The duration of hypothetical lunar atmospheres was first studied by Vondrak (1974) and reviewed by Stern (1999). For the dense atmospheres considered here (with total masses >10^8^ kg), the mass loss rate from a collisionally dominated atmosphere is expected to be ∼10 kg s^−1^, independent of atmospheric mass. The loss rate would be higher if impact erosion (Melosh and Vickery, 1989) or solar-wind stripping were important, but note that the early Moon may have been protected by a magnetic field (*e.g.,* Hood, 2011), reducing the impact of the latter process. Based on these loss rates, Needham and Kring (2017) estimated a lifetime of ∼70 million years for the transient atmosphere generated by lunar volcanic activity, and the duration of a hypothetical denser and earlier atmosphere immediately following LMO crystallization could have been even longer. In principle, liquid water could have existed on the lunar surface during these times, and perhaps even more likely in protected subsurface environments such as interstitial pore spaces within the impact-generated mega-regolith.

It is instructive to put this time frame into perspective from a natural history point of view: Lazcano and Miller (1994) speculated that the time it took from the formation of suitable building blocks of life to the first cyanobacteria was no more than 10 million years. If they are correct, the transition from a nonliving to a living system might have taken place in considerably less of a time span, perhaps as little as a few thousand years. On the other hand, Orgel (1998) has argued that we simply do not understand how life, particularly its replication system, originated on primitive Earth; thus it is not possible to come up with a reliable time estimate. If abiogenesis is able to occur on short timescales, then an origin of life on the Moon cannot be excluded. Moreover, there is an alternative scenario for introducing life to the Moon: the early history of the Solar System was dominated by giant impacts and the transfer of meteorites between planets (*e.g.,* Mileikowsky *et al.,* 2000). During this time, and indeed subsequently, it is expected that meteorites blasted off the surface of Earth will have landed on the Moon (Armstrong *et al.,* 2002; Gutiérrez, 2002; Schulze-Makuch, 2013), and some of them are expected to have survived the impact (*e.g.,* Crawford *et al.,* 2008). As life appears to have been present on Earth by 3.8–3.5 billion years ago (*e.g.,* Schopf, 1993; Schidlowski, 2001; Brasier *et al.,* 2015; Nutman *et al.,* 2016; Hassenkam *et al.,* 2017; Schopf *et al.,* 2018), and possibly by 4.1 or even 4.28 billion years ago (*e.g.,* Bell *et al.,* 2015; Dodd *et al.,* 2017), it is possible that Earth life could have inoculated transiently habitable lunar environments. We note that the chances of survival of microorganisms within terrestrial meteorites impacting the Moon would be increased by the presence of even a tenuous lunar atmosphere because this would reduce the impact velocity.

Of course, habitability requires much more than just the presence of a significant atmosphere and liquid water. Other constraints that need to be satisfied have been elaborated elsewhere (*e.g.,* Schulze-Makuch *et al.,* 2011; Cockell *et al.,* 2016). We do not know whether there were any intrinsic organic compounds on the Moon at that time, but even if not, these would likely have been delivered from Earth, carbonaceous asteroids, and perhaps other sources, via meteorite impacts (*e.g.,* Pierazzo and Chyba, 1999; Crawford *et al.,* 2008; Burchell *et al.,* 2010; Matthewman *et al.,* 2015; Svetsov and Shuvalov, 2015). Thus, sources of organics on the lunar surface may have been available. Moreover, as the early Moon appears to have had a magnetic field (*e.g.,* Hood, 2011), its surface may have been partially protected from solar and cosmic radiation.

If these early habitable environments ever existed, would there be any evidence remaining? Clearly, we do not see the familiar water-modified topography on the Moon that we see on Mars (*e.g.,* fluvial channels or crater rims that are altered by liquid water), and it is questionable whether any topographical evidence of early surface water would be preserved on the Moon after approximately 4 billion years of pounding by solar wind, cosmic radiation, and micrometeorites. On the other hand, there is some (albeit limited) evidence for oxidation and/or hydrothermal activity in lunar rocks (*e.g.,* Shearer *et al.,* 2014; Joy *et al.,* 2015). One especially promising future line of inquiry, especially for evidence of near-surface water during the high point of mare volcanism, would be to search for evidence of hydration in paleoregolith layers trapped between lava flows dating from this time (see Crawford *et al.,* 2010, for a discussion of lunar paleoregolith deposits). It is also interesting to note that Cannon *et al.* (2017) have proposed an early episode of hydration, resulting from magma ocean degassing, as the source of primordial clay mineral formation on Mars, and if such a process operated on early Mars, then it seems likely to have operated on the early Moon as well. Although it seems certain that if liquid water ever existed on the early Moon it would have been much less prevalent than on early Mars, evidence for it may yet turn up in studies of lunar samples.

Thus, if liquid water and a significant atmosphere were present on the early Moon for millions of years, it can be assumed that the lunar surface was at least transiently habitable and probably also had an inventory of the building blocks required for life. Whether life ever arose on the Moon, or was transported to it from elsewhere, is of course highly speculative and can only be addressed by an aggressive future program of lunar exploration (*e.g.,* Crawford and Joy, 2014). An important aspect of such an exploration program would be obtaining samples from paleoregolith deposits dating from time of peak mare volcanism (*i.e.,* at ∼3.5 Ga) to determine if hydrated conditions (or other evidence for habitable conditions, including possible biomarkers) existed at that time.

In addition, experiments could be conducted in lunar environment simulation chambers in laboratories on Earth to observe whether microorganisms can maintain viability under the environmental conditions predicted to have existed on the early Moon. Indeed, the surface conditions predicted by Needham and Kring (2017) are not very different from those routinely produced in Mars simulation chambers (Galletta *et al.,* 2006; Jensen *et al.,* 2008; de Vera *et al.* 2013). Such facilities are available at many research institutions around the world and could easily be modified to simulate early lunar environmental conditions. Moon-analog experiments are already done on the International Space Station (ISS) as part of the BIOMEX project (Baqué *et al.,* 2017), and methods to analyze these samples for microbial viability are being developed (Liu *et al.,* 2018). Thus, we recommend utilizing both Moon simulation chambers on our planet and on the ISS to test whether there might have been an early habitability window on the Moon.

## Notes

Associate Editor: Christopher McKay

## Abbreviations Used

ISS: International Space Station
LMO: lunar magma ocean
